# Mycology laboratory diagnostic capacity for invasive fungal diseases in public hospitals in Vietnam

**DOI:** 10.1093/mmy/myae082

**Published:** 2024-08-09

**Authors:** Vu Ngoc Hieu, Nguyen Le Hiep, Le Minh Hang, Bianca A Lau-Goodchild, Nguyen Van Duong, Nguyen Thuy Linh, Justin Beardsley, Vu Quoc Dat

**Affiliations:** Department of Microbiology, Hanoi Medical University, Hanoi, Vietnam; Hanoi Medical University Hospital, Hanoi Medical University, Hanoi, Vietnam; Hanoi University of Pharmacy, Hanoi, Vietnam; Woolcock Institute of Medical Research, Hanoi, Vietnam; Sydney Infectious Diseases Institute (Sydney ID), The University of Sydney, Darlington, NSW, Australia; Sydney School of Public Health, The University of Sydney, Camperdown, NSW, Australia; Thanh Hoa Lung Hospital, Thanh Hoa, Vietnam; Department of Administration, Hanoi Medical University, Hanoi, Vietnam; Sydney Infectious Diseases Institute (Sydney ID), The University of Sydney, Darlington, NSW, Australia; Sydney Infectious Diseases Institute (Sydney ID), The University of Sydney, Darlington, NSW, Australia; Department of Infectious Diseases, Hanoi Medical University, Hanoi, Vietnam

**Keywords:** invasive fungal infections, antifungal susceptibility testing, serology testing, molecular diagnosis

## Abstract

This was a cross-sectional study on the availability of laboratory infrastructure and capacity for the diagnosis of invasive fungal diseases in 24 public hospitals in Vietnam in 2023. Among the hospitals surveyed, 66.7% (14/21) had specialized personnel assigned for mycology testing, and 95.8% (23/24) had a separate microbiology laboratory space. Microscopy and culture methods are available in nearly all laboratories for isolate identification. Antifungal susceptibility testing is only performed for yeasts in 16/24 (66.7%) laboratories. Non-culture methods are hardly used in laboratories. Strengthening local laboratory capacities is essential to meeting health needs in these endemic regions.

## Introduction

Invasive fungal diseases (IFDs) pose a significant burden on global healthcare, resulting in an estimated 3.8 million annual deaths worldwide.^1^ This burden of disease is now being compounded by the emergence of antifungal resistance, worsening treatment outcomes, and increasing mortality rates.^2^

Accurate diagnosis is the key to effective medical treatment, and, therefore, to relieving the burden of IFDs. Laboratory diagnosis of fungal diseases includes classic microscopy and culture-based methodologies, serology, and molecular testings. The availability of all of these methods varies by geographical regions, with limited access in low- and middle-income countries^3–5^ alongside a lack of formal training in medical mycology.^6^

Vietnam has a population of 100 million and a high burden of HIV (249 000 people living with HIV in 2023) and tuberculosis (172 00 new cases in 2022)—both highly relevant to fungal infections.^7,8^ Recent estimates suggest that at least 2.5% of the population may be affected by serious fungal diseases, and this prevalence is likely underestimated due to a lack of local surveillance data and limited diagnostic capacity.^9–11^ We therefore investigated the laboratory infrastructure and diagnostic capacity for fungal diseases.

## Materials and methods

This was a cross-sectional study of public hospitals in Vietnam, conducted from July to September 2023 using a convenience sampling method. We pre-identified the largest provincial general hospitals and sent them invitations to participate in this study via paper forms.

The study questionnaire collected data on (1) hospital size, (2) mycology laboratory infrastructure, (3) capacity for fungal identification and susceptibility testing, and (4) participation in an external quality assessment (EQA) programme and research. A draft questionnaire was developed by V.Q.D., J.B., and V.N.H. and piloted with doctors from Hanoi Medical University Hospital (not included in the final analysis) before being distributed to the targeted hospitals. The forms were completed by the laboratory staff in hospitals and validated by the head of the laboratory. The collected data were transferred to an electronic database using the Qualtrics platform. Data were processed and analysed using RStudio (version 1.2.5019). Categorical data were presented as frequencies and percentages, while numerical variables were presented by median and interquartile range (IQR). Collected data were measured against the features of a model state of fungal diagnostics, as outlined in the Global Action Fund for Fungal Infections (GAFFI) roadmap and WHO Fungal Priority Pathogens List, to identify gaps for future improvement.^12,13^

## Results

A total of 24 hospitals participated in the survey, corresponding to a response rate of 40% (24/63). This included 8/24 (33.3%) central hospitals and 16/24 (66.7%) provincial/municipal hospitals. The median bed capacity of central hospitals was 1500 (IQR 1162–2000) and for provincial/municipal hospitals was 900 (IQR 637.5–1200). A total of 95.8% (23/24) had a distinct microbiology laboratory space, and 43.4% (10/23) had a separate space exclusively for mycology (Table 1).

**Table 1.** Basic characteristics and subgroup analysis by level of hospital.

Diagnostics capacity	All hospitals (*N* = 24)	Tertiary hospitals (*N* = 8)	Provincial hospitals (*N* = 16)
Laboratory structure and basic equipment			
Separate microbiology space	23/24 (95.8%)	8/8 (100%)	15/16 (93.8%)
Separate mycology space	10/24 (41.7%)	4/8 (50%)	6/16 (37.5%)
Separate mycology biosafety cabinet	11/24 (45.8%)	4/8 (50%)	7/16 (43.8%)
Separate mycology incubator	10/24 (47.6%)	3/8 (37.5%)	7/16 (43.8%)
Only 30°C	1/24 (4.2%)	1/8(12.5%)	0
Only 37°C	6/24 (25%)	0	6/16 (37.5%)
Both 30°C and 37°C	3/24 (12.5%)	2/8 (25%)	1/16 (6.25%)
External quality assessment and research			
Participation in EQA schemes	4/24 (16.7%)	2/8 (25%)	2/16 (12.5%)
National level	1/24 (4.2%)	0	1/16 (6.25%)
Provincial level	1/24 (4.2%)	0	1/16 (6.25%)
Storage of fungal isolates			
Yeast			
No	16/24 (66.7%)	3/8 (37.5%)	13/16 (81.3%)
<1 year	3/24 (12.5%)	2/8 (25%)	1/16 (6.25%)
≥1 year	5/24 (20.8%)	3/8 (37.5%)	2/16 (12.5%)
Mould			
No	19/24 (79.2%)	6/8 (75%)	13/16 (81.3%)
<1 year	2/24 (8.3%)	1/8 (12.5%%)	1/16 (6.25%)
≥1 year	3/24 (12.5%)	1/8 (12.5%)	2/16 (12.5%)

EQA, external quality assessment.

Few laboratories were involved in research, and storage of isolates was uncommon. Only 20.8% (5/24) indicated that they stored yeast isolates for longer than one year, dropping to 12.5% (3/24) for moulds (Table 1). Subgroup analysis suggested that laboratories interested in research were more likely to store clinical isolates. Among 12 hospitals with reports of fungal isolates in 2022, the most commonly identified pathogens were *Candida* spp. (including 1288 *C. albican* isolates, 227 *C. tropicalis* isolates, 126 *C. parasilosis* isolates, 8 *C. auris isolates*, and 8 *Candida* spp. isolates), *Aspergillus fumigatus* (24 isolates), and *Cryptococcus neoformans* (9 isolates) (data not shown). A detailed breakdown of the availability of different diagnostic tests ‘on-site’ is shown in Table 2.

**Table 2.** Diagnostic services available on-site.

Diagnostic services	All hospitals (*N* = 24)	Tertiary hospitals (*N* = 8)	Provincial hospitals (*N* = 16)
Microscopic examination	24/24 (100%)	8/8 (100%)	16/16 (100%)
KOH	23/24 (95.8%)	8/8 (100%)	15/16 (93.7%)
India ink stain	9/24 (37.5%)	4/8 (50%)	5/16 (31.25%)
Others	0	0	0
Identification of fungi			
Yeast			
Blood culture	18/24 (75%)	7/8 (87.5%)	11/16 (68.7%)
Fungal culture of other samples	16/24 (66.7%)	7/8 (87.5%)	9/16 (56.2%)
CHROMagar *Candida*	7/24 (29.2%)	4/8 (50%)	3/16 (18.7%)
API 20C AUX or ID32c	2/24 (8.3%)	1/8 (12.5%)	1/16 (6.25%)
Vitek 2 system	14/24 (58.3%)	7/8 (87.5%)	7/16 (43.7%)
MALDI-TOF	4/24 (16.7%)	3/8 (37.5%)	1/16 (6.25%)
Mould			
Morphological characterization	16/24 (66.7%)	7/8 (87.5%)	9/16 (56.2%)
Others	0	0	0
Antifungal susceptibility testing	16/24 (66.7%)	8/8 (100%)	8/16 (50%)
Vitek 2 system	13/24 (54.2%)	6/8 (75%)	7/16 (43.7%)
Disk diffusion	2/24 (8.3%)	2/8 (25%)	0
Microdilution broth	3/24 (12.5%)	2/8 (25%)	1/16 (6.25%)
Yeast one	6/24 (25%)	4/8 (50%)	2/16 (12.5%)
Etest	1/24 (4.1%)	1/8 (12.5%)	0
Serology testing (*N* = 24)			
β-D glucan test	1/24 (4.1%)	1/24 (4.1%)	0
Galactomannan test	1/24 (4.1%)	1/24 (4.1%)	0
Aspergillus IgG test	1/24 (4.1%)	1/24 (4.1%)	0
Molecular testing (*N* = 24)	5/24 (20.8%)	3/8 (37.5%)	2/16 (12.5%)
*Candida* PCR	2/24 (8.3%)	2/8 (25%)	0
Other PCR	0	0	0

MALDI-TOF, matrix-assisted laser desorption/ionization time-of-flight; IgG, immunoglobulin G; PCR, polymerase chain reaction.

The main methods for identification of yeasts were biochemical phenotypic methods using automatic instruments (58.3%), followed by chromogenic agar media for rapid *Candida* identification (29.2%), and benchtop biochemical methods like API AUX or ID (8.3%). The identification of moulds was only based on the morphological characterization.

Antifungal susceptibility testing was conducted in 66.7% of laboratories, but was only performed for yeast. The VITEK automated system was used by 54.2% of laboratories (13/24), followed by Sensititre YeastOne (*n* = 6, 25%), broth microdilution (*n* = 3, 12.5%), disk diffusion (*n* = 2, 8.3%), and Etest (*n* = 1, 4.1%) (Table 2). None laboratories refer samples to other hospitals or a third party for susceptibility testing.

Relative to an ideal fungal diagnostic system, prominent gaps identified across participant hospitals included a lack of laboratories with spaces exclusively dedicated towards mycology testing, lack of antifungal susceptibility testing, and an absence of serological and molecular tests. Limited use of India ink staining for cryptococcal diagnosis, MALDI-TOF MS, and PCR was identified as important gaps inhibiting access to timely and accurate fungal diagnosis. Further, limited participation in external mycology quality assessment services, limited participation in research activities, and limited demand for education and training to promote fungal diagnosis capacity were notable gaps.

## Discussion

In Vietnam, fungal infections pose a significant public health concern, with the majority of the burden being attributable to tuberculosis and HIV/AIDS.^11^ Here, we present the first national survey on the capacity for IFD diagnostics, which adds significant depth to the previous study by Salmonton-Garcia et al.^4^

Accurate identification of fungal species is important for selection of suitable antifungal therapy. In 2022, WHO published the Fungal Priority Pathogens List, calling for action to improve the prevention, diagnosis, and treatment of these priority pathogens. The GAFFI list of core diagnostic tests that should be present in a reference lab, including direct microscopy, antigen detection, molecular tests, fungal culture, and identification, forms the basis of our recommendations.^12^

An ideal fungal diagnostic system offers access to affordable diagnostic tests for fungal infections for timely and accurate diagnosis, incorporating antifungal susceptibility testing. It should support ongoing surveillance to inform clinical practice, helping clinicians screen for and treat life-threatening IFDs to improve patient survival.^3^ Our findings revealed a significant departure from this model system of fungal diagnostic capacity, with all of the participating laboratories lacking sufficient fungal testing capability to act as independent mycology laboratories, with a particularly notable lack of essential antigen detection and molecular tests.^12,13^

Another major issue was the lack of participation in regular external mycology quality assessment. This is a useful marker of competence of a laboratory,^14^ ensuring patients have access to high quality diagnostics.^5^

The disproportionate burden of IFDs on immunocompromised individuals, increasing emergence of MDR fungal species with their detrimental impacts on treatment outcomes and mortality, and the growing rate of at-risk populations stress the importance of reliable health system capacity for fungal disease diagnosis. There is a pressing need to close this gap between the current national system’s capacity for fungal diagnosis and the ideal standard. Timely access to accurate and quality fungal diagnostics promotes the rational use of antifungal agents for effective treatment and reduces unnecessary empiric antimicrobial use in line with the WHO agenda on antimicrobial resistance.^13,15^

This first national survey on IFD diagnostics reveals substantial diagnostic gaps in Vietnam. None of the labs surveyed could perform the full range of ‘core tests’ outlined by GAFFI. Participation in external mycology quality assessment programmes was relatively low compared to other Asian countries. It is evident that laboratory capacity is not currently keeping pace with the growing disease burden of IFDs.
